# Complementary music therapy for cancer patients in at-home palliative care and their caregivers: protocol for a multicentre randomised controlled trial

**DOI:** 10.1186/s12904-020-00570-9

**Published:** 2020-05-02

**Authors:** Inmaculada Valero-Cantero, Francisco Javier Martínez-Valero, Milagrosa Espinar-Toledo, Cristina Casals, Francisco Javier Barón-López, María Ángeles Vázquez-Sánchez

**Affiliations:** 1 Puerta Blanca Clinical Management Unit, Malaga-Guadalhorce Health District, Malaga, Spain; 2 Midlothian Foot Care, Dalkeith and National Health Service, Lothian, Scotland; 3 “Rincón de la Victoria” Clinical Management Unit, Malaga-Guadalhorce Health District, Malaga, Spain; 4 MOVE-IT Research group and Department of Physical Education, Faculty of Education Sciences. Biomedical Research and Innovation Institute of Cadiz (INiBICA) Research Unit, Puerta del Mar University Hospital, University of Cadiz, Cádiz, Spain; 5 Department of Preventive Medicine, Public Health and Science History. Institute of Biomedical Research in Malaga (IBIMA), University of Malaga, Malaga, Spain; 6 Department of Nursing, Faculty of Health Sciences, University of Malaga, Malaga, Spain

**Keywords:** Patient care, Oncology, Palliative therapy, Home care, Family caregiver, Quality of life, Nurses, Cost efficiency analysis

## Abstract

**Background:**

Patients with advanced cancer, receiving at-home palliative care, are subject to numerous symptoms that are changeable and often require attention, a stressful situation that also impacts on the family caregiver. It has been suggested that music therapy may benefit both the patient and the caregiver. We propose a study to analyse the efficacy and cost utility of a music intervention programme, applied as complementary therapy, for cancer patients in palliative care and for their at-home caregivers, compared to usual treatment.

**Method:**

A randomised, double-blind, multicentre clinical trial will be performed in cancer patients in at-home palliative care and their family caregivers. The study population will include two samples of 40 patients and two samples of 41 caregivers. Participants will be randomly assigned either to the intervention group or to the control group. The intervention group will receive a seven-day programme including music sessions, while the control group will receive seven sessions of (spoken word) therapeutic education. In this study, the primary outcome measure is the assessment of patients’ symptoms, according to the Edmonton Symptom Assessment System, and of the overload experienced by family caregivers, measured by the Caregiver Strain Index. The secondary outcomes considered will be the participants’ health-related quality of life, their satisfaction with the intervention, and an economic valuation.

**Discussion:**

This study is expected to enhance our understanding of the efficacy and cost-utility of music therapy for cancer patients in palliative care and for their family caregivers. The results of this project are expected to be applicable and transferrable to usual clinical practice for patients in home palliative care and for their caregivers. The approach described can be incorporated as an additional therapeutic resource within comprehensive palliative care. To our knowledge, no previous high quality studies, based on a double-blind clinical trial, have been undertaken to evaluate the cost-effectiveness of music therapy. The cost-effectiveness of the project will provide information to support decision making, thereby improving the management of health resources and their use within the health system.

**Trial registration:**

The COMTHECARE study is registered at Clinical Trials.gov, NCT04052074. Registered 9 August, 2019.

## Background

Cancer presents a huge challenge to global public health, and remains one of the leading causes of morbidity and mortality worldwide [1]. According to a 2018 report by the Spanish Society of Medical Oncology, based on data published by the National Statistics Institute (INE), in 2016 almost 113,000 people died in Spain of cancer-related causes (27.5% of deaths). This figure was 1.4% higher than in 2015 and by 2035 it is expected to reach almost 160,000 [2]. These statistics are similar to those reported elsewhere [3], reflecting the fact that despite the great advances achieved in cancer treatment, levels of comorbidity and mortality remain high, and in many cases palliative care is the only resource available [4].

The World Health Organization defines palliative care as “an approach that improves the quality of life of patients and their families facing the problem associated with life-threatening illness, through the prevention and relief of suffering by means of early identification and impeccable assessment and treatment of pain and other problems, physical, psychosocial and spiritual” [5].

It is important to realise that palliative care should not be limited to the last days of life, but provided progressively during the course of the disease, in accordance with the needs of the patient and family members. Among other aspects, it should include relief from pain and other symptoms, together with attention to spiritual and psychological questions, helping patients live as actively as possible until death and providing support to help the family adapt, both during the illness and in bereavement [5, 6].

Common problems in patients with advanced cancer include lack of appetite, nausea, vomiting, constipation, diarrhoea, dyspnoea, insomnia, pain, anxiety, depressive symptoms and fatigue [7, 8]. Moreover, these symptoms appear to be interrelated. Thus, studies have shown that anxiety, fatigue, depression, anxiety, uncertainty and hopelessness may interact with pain [9, 10]. Accordingly, if one or more of these conditions is alleviated, others may be expected to improve, too.

For their part, family caregivers are subjected to constant stress, which in many cases is greater than that experienced by other informal caregivers, since the patient often presents severe and changing symptoms that require close, constant care and attention. Under these conditions, the physical health of the caregiver may be affected [11, 12], with a consequent negative impact on their quality of life [13, 14].

The incorporation of music therapy as part of the palliative care might help patients and caregivers in managing pain and anxiety, enhancing their mood, promoting relaxation, facilitating the expression and channelling of emotions, and offering a measure of support during grieving. Diverse theories have been proposed to explain how the human brain processes emotions. One such refers to the classical subcortical route, in which the limbic system plays a fundamental role [15]. The type of melody employed in a given piece of music is known to influence the listener, who may recognise its nature, for example, as happy or sad. In identifying a melody according to its emotional nature, the lower frontal gyrus, the medial thalamus and the dorsal anterior cingulate may all play significant roles [16].

Music that produces intense pleasure or excitement activates the neural systems of reward and emotion in a similar way to other biologically relevant stimuli, such as food, sex or psychoactive drugs [17]. The ability of music to induce pleasure and to stimulate endogenous reward systems suggests that, although it may not be essential for the survival of the human species, music greatly benefits our mental and physical well-being [17].

Nursing Interventions Classification (NIC) 4400 defines music therapy as the use of music to help achieve a specific change in patients’ behaviour, feelings or physiology [18]. Research findings suggest that music can benefit cancer patients in palliative care in areas such as alleviating pain [19] or depression [20, 21], meeting psychosocial needs [22] and enhancing the quality of life [23]. Moreover, there is a synergy between the therapeutic aims of music therapy and those of palliative care; thus, a significant proportion of the participants in a study conducted in this field perceived music therapy to be effective [24]. However, another study reported that although music seems to benefit patients, it sometimes reminds them of their altered state [25].

Various systematic reviews on the use of music therapy have concluded that it can produce beneficial effects, reducing pain [26–28], anxiety, pain and fatigue and enhancing the quality of life [29, 30]. However, these reviews have also observed that many trials in this area are subject to bias and that more high quality research is required. Music therapy is appreciated by patients and others, because it promotes social interaction between patients and those around them, together with the sensation that more comprehensive care is being provided, meeting not only physical needs, but also psychological and spiritual ones [31, 32]. Few studies have been undertaken to consider the influence of music therapy on the well-being of caregivers of patients in palliative care. Nevertheless, researchers have observed an immediate positive impact of music therapy [33–35], and other studies have suggested it may be beneficial during grieving [36].

The provision of music therapy could also be financially beneficial, through associated decreases in spending, especially as concerns the reduced need for medication [37, 38]. Savings may also be achieved as less time and/or fewer medical staff are required to provide attention [37, 38]. However, these conclusions were not drawn from studies focused specifically on palliative care patients. Regarding the impact of music therapy on the organisation of health services, despite its potential benefit in areas such as optimising the provision of medication and reducing the consumption of healthcare resources [37, 38], very limited evidence has been offered regarding the cost-effectiveness of this type of intervention. Therefore, in addition to evaluating the effectiveness of music therapy, we will also determine its efficiency, by means of a specific economic evaluation method, thus helping decision makers optimise the use of health resources and providing information on the economic impact of the intervention.

The present study will consider the use of music therapy both for patients and for their caregivers, taking into account that a particular style of music may present a wide range of options. In other words, the same music does not activate emotions in the same way among different listeners, because on many occasions it is associated with personal experience. Nevertheless, most people can distinguish between sad and happy melodies; we all experience certain types of music that energise us or make us sad, and the listening choice made depends on personal preference. In view of these considerations, we assume that benefits are derived from listening to the music that pleases the individual concerned, as is essential if positive results are to be obtained from music therapy. To avoid or minimise bias, this clinical trial will be conducted in accordance with all the recommendations made in the 2016 Cochrane review [29].

The overall aim of the study described is to evaluate the efficacy and cost-utility of a programme of musical intervention applied as complementary therapy for cancer patients in palliative care and for their caregivers in the home setting, compared to usual treatment. The participants’ satisfaction with the intervention will also be assessed.

## Methods / design

### Study design

The study will take the form of a randomised controlled trial (RCT), double blinded and multicentre, with a study population composed, on the one hand, of cancer patients receiving at-home palliative care and, on the other, of their informal caregivers. In each case, two parallel groups will be considered: a control group (CG) and an experimental group (EG).

The study will be conducted at six Primary Care Clinical Management Units: Puerta Blanca, Tiro de Pichón, Nueva Málaga, La Luz, Palma-Palmilla and Rincón de la Victoria Basic Zone, all within the Málaga-Guadalhorce Health District.

### Patients and recruitment

Patients will be recruited to this study from the lists of cancer patients included in the Palliative Care Attention Process in the DIRAYA Digital Health History, corresponding to one of the Clinical Management Units participating in the study. These patients’ informal caregivers will also be recruited.

**Fig. 1:**
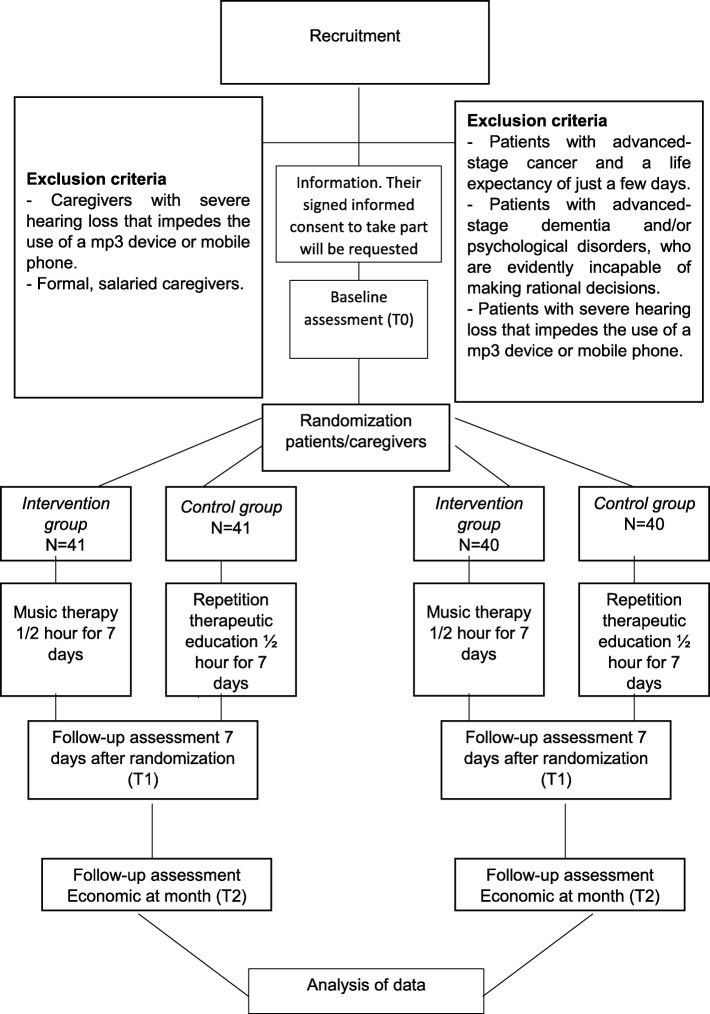
Flow chart for the Randomized Controlled Trial

A list of random numbers supplied by the Epidat 4.2 program will be used to select the patients in palliative care required for this study. These patients will be contacted and assessed to determine their suitability for inclusion. If the criteria are met, the patients will be invited to participate and given an information leaflet with full details of the procedure. Their signed informed consent to take part will be requested. When the patient has an informal caregiver, this person will also be invited to join the study, given the information leaflet and asked to sign the informed consent form. The flow chart for the RCT is shown in Fig. 1.

### Inclusion criteria

- Cancer patients receiving at-home palliative care, aged over 18 years, and their informal caregiver (if any).
- Provision of signed informed consent to participate.

### Exclusion criteria

- Patients with advanced-stage cancer and a life expectancy of just a few days.
- Patients with advanced-stage dementia and/or psychological disorders, who are evidently incapable of making rational decisions.
- Patients with severe hearing loss that impedes the use of a mp3 device or mobile phone.
- Caregivers with severe hearing loss that impedes the use of a mp3 device or mobile phone.
- Formal, salaried caregivers.

### Sample size

The most significant variable in palliative care patients is assumed to be anxiety. In a review conducted by Bradt et al. [29], this variable presented a distribution with a standard deviation of 1.8, for a population similar to that considered in the study we propose, albeit with a wider range of results. Previous studies have reported that music therapy has a significant impact on this variable. In one case, an effect size of 7 was measured (effect size of 29% of the standard deviation). Another study, of cancer patients receiving treatment including ginseng [39], recorded a variation of 0.83 points on the Edmonton scale, with a standard deviation of 2.34 (35% of the standard deviation). Assuming a 2-point decrease in before-after anxiety to be clinically relevant, and a maximum standard deviation of 2.5 in our population, with an alpha error of 0.05 and a beta of 0.10, we calculate that two samples of 33 subjects will be necessary, to enable the option of a 1:1 ratio between the samples. For convenience, and to allow for losses to follow up, this sample size will be increased by 20%, to two samples of 40 persons. The Epidat 4.2 statistical program was used to perform these calculations.

Hanser et al. [40] recorded an increase in caregivers’ satisfaction following the incorporation of music therapy into the healthcare provided, with a standard deviation of 1.33 for relaxation, 1.87 for comfort, and 1.23 for happiness. Taking into account these findings and assuming an increase of one point in the dimension of caregiver happiness to be clinically relevant, we calculate that two samples of 34 caregivers will be necessary. For convenience, this sample size will be increased by 20%, to two samples of 41 persons, to allow for losses to follow up.

### Control of bias

In order to avoid bias, the participants will be randomly assigned to the study groups (EG or CG). They will not be told which group they have been assigned to. Moreover, they will be blinded to the procedure, as headphones will be provided to both groups; the intervention group will listen to music, while the control group will listen to (spoken word) messages of therapeutic education via an mp3 player or mobile phone. The evaluators will also be blinded to the assignation of groups. The statistical analysis will be based on intention to treat.

### Intervention protocol

#### Control group

The usual treatment will be provided, in accordance with the Palliative Care Plan of the Andalusian Ministry of Health [41] and with the rules and recommendations for Palliative Care Units issued by the Ministry of Health and Social Policy [42]. This treatment consists of an initial comprehensive assessment by an attending nurse, both of the person in palliative care and of their caregiver, by reference to the Virginia Henderson of 14 basic human needs, together with application of the care plan designed for the patient and periodic observation of any problems or symptoms that may arise. In addition, basic therapeutic education will be provided on questions such as food and hydration, exercise and leisure, medication, effective communication, skin care, the prevention and treatment of constipation, and sleep hygiene. In order to mask the membership of the study group (intervention or control), the patients assigned to the control group will again receive the basic therapeutic education previously supplied in a conventional way, but this time via an mp3 player or mobile phone, using an audio file available on Google Drive), in seven 30-min daily sessions. This procedure also ensures the blinding of the case management nurse responsible for evaluating the patients’ condition.

#### Intervention group

These persons will receive not only conventional health care, but also music therapy, provided via pre-recorded music (according to personal taste), both to the palliative care patients and to their caregivers. Those assigned to this group will be advised to select music that enhances their well-being. The music will be supplied via an mp3 player or mobile phone (using the Spotify Premium program, which is available without charge for a one-month trial period), in seven 30-min daily sessions.

### Blinding

The only person aware of the groups to which patients and their caregivers have been assigned will be the researcher responsible for the randomisation. Neither the evaluators (case management nurses) nor the study subjects will know which group they are in. This masking will be achieved by providing headphones, for use with an mp3 player or mobile phone, to the participants in both groups, who will be instructed not to tell the evaluator what they are listening to. Thus, none of the participants will know whether they belong to the control group or to the intervention group.

### Randomisation

The following randomisation mechanism will be applied: cards marked “intervention group” or “control group” will be prepared, in quantities corresponding to the numbers required for each group. These cards will be placed in sealed opaque envelopes, which will be shuffled and then numbered, such that none of those involved will know the group to which each envelope corresponds. Each of the participants will be assigned a participation number (by order of entry to the study). The researcher responsible for the randomisation will open the envelope when the patient/caregiver confirms their intention to participate. This system ensures that the group sizes foreseen will be achieved and that the assignation to the study groups is unlikely to be manipulated.

Each eligible patient in palliative care and, if applicable, their caregiver will be invited to participate in the study. If they both accept, a single envelope will be used for both persons (i.e. both will be in the same group, control or intervention). If only one accepts, they will also be given an envelope assigning them to the control or the intervention group. Participants will continue to be assigned until the pre-established sample size is reached.

When the researcher performing the randomisation opens the envelope and is aware of which group the patient (and caregiver, if applicable) belongs to, they will be informed of the corresponding procedure that will be applied, without mention of whether this refers to a control or an intervention group. The same researcher will instruct the participant in the use of the mp3 player to access an audio file through Google Drive or in the use of the Spotify app on the mobile phone, as the case may be. The participant will also be asked not to perform any activity other than listening during the intervention, and not to let the evaluator know what they have heard, explaining that this restriction will help make the research data more reliable.

If the patient is a member of the intervention group, in addition to the above, the researcher will ask them to make a list (at home) of 15 songs or musical items that put them in a good mood, and to send it (by mail or by phone) to the researcher so that the titles selected can be obtained and made ready for the beginning of the sessions, available either on the mp3 player or through the music app of the mobile phone. Participants in the control group will listen to health education advice, on the mp3 player or via Google Drive on their mobile phone.

The case management nurses at the six healthcare centres collaborating with this study will evaluate the study variables at three time points: 1 day before the participant receives the music delivery system from the researcher, 7 days after the intervention and 30 days after the intervention. These nurses will be blinded to the study group to which the participant is assigned.

### Outcome assessment

The primary outcome measures that will be considered to assess the effectiveness of the intervention are the symptoms presented by the patients and the degree of overload experienced by the caregivers. The secondary outcomes considered, for patients and caregivers, are health-related quality of life, satisfaction with the intervention and the economic valuation. The outcome measures will be obtained from questionnaires completed at the start of the study (t0), at 1 week after randomisation (t1) and at 1 month after the start (t2).

### Primary outcome measure. Variables and measurement instruments for patients

Patients’ symptoms will be evaluated by the Edmonton Symptom Assessment System (ESAS), validated in Spanish [43]. This instrument considers ten frequently-occurring symptoms in cancer patients – pain, tiredness, nausea, depression, anxiety, drowsiness, dyspnoea, anorexia, sleep disorders and malaise – that may have been experienced by the patient during the previous week. The patient is asked to select the number that best indicates the intensity of each symptom, on a scale ranging from zero to ten (0 = no symptom, 10 = maximum severity). The ESAS symptoms are then classified as the physical component (pain, shortness of breath, lack of appetite, nausea, tiredness and drowsiness) and the psychological component (anxiety and depression), which are combined to produce the overall ESAS score.

### Secondary outcome measures. Variables and measurement instruments for patients

- Sociodemographic data for patients in palliative care: age, sex, marital status, level of education, type of cancer and time spent in palliative care.
- Health-related quality of life will be assessed according to the 30-item core European Organisation for Research and Treatment of Cancer Quality of Life Questionnaire (EORTC QLQ C30), version 3.0, validated in Spanish [44]. In this instrument, items 1–28 are based on mixed categorical scales ranging from 1 to 4 and refer to the patient’s functional status, the presence of physical and emotional symptoms and their impact on work and socio-family life. Items 29 and 30 assess general aspects of the patient’s health and quality of life, on a scale ranging from 0 to 7. This scale is used to assess the quality of life of patients in palliative care.
- Quality-Adjusted Life Year (QALY). This parameter is obtained according to the European Quality of Life-5 Dimensions-5 Levels (EuroQol-5D-5 L) questionnaire [45], validated in Spanish [46], which describes the patient’s health status in five dimensions (mobility, personal care, usual activities, pain/discomfort and anxiety/depression). Results are also obtained on a visual analogue scale (VAS), graduated from 0 to 100, representing the range from “worst imaginable health status” to “best imaginable health status”, respectively.
- Use made of health resources: number of consultations in primary care (family doctor and family nurse), number of home visits (family doctor, family nurse and case manager), number of consultations made to the primary care emergency service, number of consultations made to the hospital emergency department and number of hospital admissions (recording the number of days of admission). The time spent by health care personnel in responding to telephone calls to the palliative care unit and to the case manager will also be taken into account.
- Consumption of medication - analgesics, anxiolytics and sleeping pills.
- Willingness to pay. The patients’ willingness and ability to pay for musical therapy will be evaluated using the contingent valuation method [47] in which, in a hypothetical scenario, patients are asked whether they would be prepared to pay for music therapy.
- Satisfaction with palliative care. The patient’s satisfaction with the care received will be determined using the CSQ-8 Client Satisfaction Questionnaire, a self-administered instrument consisting of 8 questions, evaluated on a 4-point Likert scale, with specific cut-off points for each item. The questionnaire focuses on the quality and type of service received, the results achieved and overall satisfaction. Also included are three open questions, inviting the respondent to describe the best and worst aspects of the service received, and the changes that should be made. This instrument determines patient satisfaction with high reliability and consistency, and has been validated, in its 8-item version, for use with a Spanish-speaking population [48]. This version, too, provides high internal consistency and concurrent validity.

### Primary outcome measure. Variables and measurement instruments for caregivers

- Caregiver overload is assessed by the Caregiver Strain Index (CSI) [49], using a questionnaire translated and adapted for a Spanish-speaking population [50]. The CSI is designed for caregivers of dependent people in general, not specifically those receiving palliative care. The format used is that of a semi-structured interview, consisting of 13 true-false items. Each affirmative answer is scored as 1 point and a total score of 7 or more points is indicative of a high level of strain.

### Secondary outcome measures. Variables and measurement instruments for caregivers

- Sociodemographic data for caregivers: age, sex, marital status, level of education, working outside home (yes/no), time spent providing daily care, help received in providing care, total time spent providing care, and relationship with the person receiving care.
- The quality of life of the caregiver will be assessed by the Family Quality of Life (FQOL) scale [51], which measures the quality of life of a family member caring for a patient with cancer. The possible scores on this scale range from 0 = worse result to 10 = best result, and the total score possible ranges from 0 to 100. Some of the items are awarded an inverse score, and so when the results are tallied and coded, the scores for those elements must be reversed. The FQOL, which has been validated for use with a Spanish-speaking population [52], addresses physical, psychological, spiritual and social aspects of the caregiver’s experience.
- QALY.
- Use made of health resources: similar to the assessment made in this respect of the patient receiving palliative care.
- Consumption of medication: similar to the assessment made in this respect of the patient receiving palliative care.
- Willingness to pay: similar to the assessment made in this respect of the patient receiving palliative care.
- Satisfaction, CSQ-8.

An overview of the primary and secondary outcome measures is shown in Table 1.

**Table 1:** Measurement overview

Patient		T0	T1 7 days	T2 30 days
Primary outcome	Edmonton Symptom Assessment System (ESAS)	X	X	
Secondary outcomes	The Quality of Life of Cancer Patients (EORTC. QLQ-30)	X	X	
	Quality-Adjusted Life Year (QALY)	X	X	
	EuroQol-5D-5 L	X	X	
	Variables cost-utility analysis			
	Health Resources Consumption	X	X	X
	Medication consumption (analgesics, anxiolytics, sleeping pills)	X	X	X
	Availability to pay		X	
	Client Satisfaction Questionnaire (CSQ-8)		X	
	Other variables			
	Sociodemographic data: age, sex, marital status and level of education.	X		
	Palliative care time	X		
	Type of cancer	X		
**Caregiver**		**T0**	**T1 7 days**	**T2 30 days**
Primary outcome	Caregiver Strain Index (CSI),	X	X	
Secondary outcomes	The Quality of Life Family Version (QOL)	X	X	
	Quality-Adjusted Life Year (QALY)	X	X	
	EuroQol-5D-5 L	X	X	
	Variables cost-utility analysis			
	Health Resources Consumption	X	X	X
	Medication consumption (analgesics, anxiolytics, sleeping pills)	X	X	X
	Availability to pay		X	
	Client Satisfaction Questionnaire (CSQ-8)		X	
	Other variables			
	Sociodemographic data: age, sex, if you work away from home, marital status and level of education.	X		
	Time spent on care	X		
	Care support	X		
	Caring time	X		
	Relationship with the caregiver	X		

### Statistical analysis

A descriptive study will be made of the variables collected, calculating the mean and standard deviation in each case for the normally-distributed continuous variables, the confidence interval as appropriate for specific estimates, the median and interquartile range for non-normally-distributed continuous variables, and frequencies and percentages for categorical variables. The normality of the distribution will be determined by the Shapiro-Wilk test. Baseline values for the two groups will be compared.

The before and after-intervention values, in the control and intervention groups, will be compared by Student’s t-test for related samples in the case of normal continuous variables, and by the Wilcoxon T-test for paired data in the case of non-normal continuous variables.

In addition to this bivariate analysis, a multiple linear regression will be performed, in which the following dependent variables will be addressed, depending on the case: pain, fatigue, nausea, depression, anxiety, drowsiness, dyspnoea, anorexia, sleep disorders, malaise/well-being and quality of life. The independent variables considered will include both the intervention itself and also the participants’ sociodemographic variables (sex, age, level of education, marital status) and clinical variables (pathology, total time receiving palliative care). A similar procedure will be followed to determine the significance of the consumption of analgesics and the number of visits made by the nurse. All results obtained will be presented at a confidence interval of 95%. Thus, *p* < 0.05 is assumed to be statistically significant. The statistical software SPSS 23 and Epidat 4.2 will be used in this analysis.

### Economic evaluation

The economic evaluation performed will be a cost-utility analysis, following previous recommendations in this respect for the analysis of healthcare technologies [53]. For each group, the cost, the incremental cost, the QALY effectiveness, the incremental effectiveness and the dominance will be calculated. If there is no dominance, the final results will be expressed in terms of the incremental cost-utility ratio.

Both direct health resources costs and direct intervention-related costs will be considered. The former will be determined according to the public prices reported by the Andalusian Public Health System [54]. The cost of the direct resources incurred in the intervention will be estimated according to the corresponding retail prices. The EuroQol-5D-5 L questionnaire will be used to calculate the QALY variable. The variables subject to uncertainty will be subjected to univariate and multivariate sensitivity analyses.

## Discussion

We present the protocol for a double-blind multicentre randomised controlled trial of the effects of music therapy. The main aim of this project is to employ cost-utility analysis to evaluate the effectiveness and cost-effectiveness of a musical therapy intervention (as a complementary therapy) for cancer patients and their caregivers in at-home palliative care, compared with usual treatment.

The principal strength (and novel aspect) of this study resides in the fact that in the intervention, each participant chooses the music they believe will enhance their well-being. This contrasts with most other studies in this field, where the music is predetermined or grouped into broad categories.

Another novel aspect in the study design is the use of the double-blind system to reduce bias and maximise the reliability of the results obtained. In addition, we aim to provide new evidence on the cost-effectiveness of this kind of intervention (to our knowledge, virtually no evidence has previously been reported in this regard).

The study proposed will improve our understanding of how music therapy impacts on the physical and psychological situations of patients and their caregivers.

Potential benefits of musical therapy include the decreased consumption of medication for problems such as anxiety, pain and sleeplessness, the alleviation of symptoms, improved mood, reduced caregiver strain, enhanced health-related quality of life and greater patient and caregiver satisfaction.

As the intervention has no side effects, it can be applied in conjunction with usual short-term clinical practice, if its effectiveness is demonstrated. This type of complementary therapy could benefit the sustainable implementation and maintenance of the at-home palliative care provided to cancer patients, and at the same time improve the quality of life of their caregivers.

## Data Availability

Not applicable; data sharing is not applicable to this article as no datasets were generated or analysed yet. The preliminary datasets generated during the study are not publicly available due it has not been completed but will be available from the corresponding author on reasonable request.

## Abbreviations

CG: Control group
CSI: Caregiver Strain Index
CSQ-8: Client Satisfaction Questionnaire
EG: Experimental group
EORTC QLQ C30: 30-item core European Organization for Research and Treatment of Cancer Quality of Life Questionnaire
ESAS: Edmonton Symptom Assessment System
EuroQol-5D-5 L: European Quality of Life-5 Dimensions-5 Levels
FQQL: The Quality of Life Family Version
NIC: Nursing Interventions Classification
QALY: Quality-Adjusted Life Year
RCT: Randomized controlled trial
VAS: Visual Analogue Scale
